# Avoiding aggregation of human bone marrow–derived mesenchymal stem cells stored in cell preservation solutions

**DOI:** 10.1007/s11626-024-00849-8

**Published:** 2024-02-15

**Authors:** Takeshi Kikuchi, Masuhiro Nishimura, Chikage Shirakawa, Yasutaka Fujita, Takeshige Otoi

**Affiliations:** 1 Research and Development Center, Otsuka Pharmaceutical Factory, Inc., 115 Kuguhara, Tateiwa, Muya-cho, Naruto, Tokushima, 772-8601 Japan; 2 Bio-Innovation Research Center, Tokushima University, 2272-2 Ishii, Myozai-gun, Tokushima, 779-3233 Japan

Mesenchymal stem cells (MSCs) are widely used in clinical practice, most commonly administered intravascularly (Moll *et al.* 2019). Rapid, high-dose administration of cells into blood vessels increases the risk of vascular embolization; thus, intravascular cells are administered slowly over a period of approximately 1 h (Adas *et al.* 2021). Cells are typically administered at room temperature, and the cells are often suspended in a saline solution with or without dextrose (Riordan *et al.* 2018; Adas *et al.* 2021; Dilogo *et al. *2021; Murata *et al.* 2021). We considered that saline solution is not an ideal medium for cell administration performed slowly at room temperature; therefore, we developed several improved cell preservation solutions, namely, lactated Ringer’s solution containing 3% trehalose (LR-3T) and lactated Ringer’s solution containing 3% trehalose and 5% dextran 40 (LR-3T-5D). LR-3T maintains cell viability during storage for up to 24 h under refrigeration or at room temperature. The risk of vascular embolization is thought to increase during rapid cell administration. As sedimentation of cells in the infusion line can increase the rate of cell administration, it is necessary to equalize the cell concentration throughout the suspension. Dextran, a component of LR-3T-5D, suppresses cell sedimentation and thereby helps maintain a stable concentration of cells in the infusion line (Fujita *et al.* 2020). This solution was shown to improve the preservation of porcine embryos (Lin *et al.* 2022a; Lin *et al.* 2022b), and the addition of dimethyl sulfoxide or propylene glycol allows solutions to be used for cryopreservation (Fujita *et al.* 2021) of, for example, porcine bone marrow–derived MSCs (Nishimura *et al.* 2019; Kikuchi *et al.* 2021).

Aggregates in blood vessels can cause embolization in microvessels (Yaykasli *et al.* 2021); thus, cells administered into blood vessels must be non-aggregated. The risk of embolization is particularly high in the lungs due to the high number of microvessels. The efficacy of intravascular administration of MSCs for treating coronavirus infectious disease–2019 (COVID-19)–related acute respiratory distress syndrome was recently reported (Qu *et al.* 2022), heightening the importance of obtaining safety information on the aggregation of administered cells.

In this study, we investigated the conditions under which cell aggregation occurs and methods for suppressing this aggregation by varying preservation time, temperature, and trypsin treatment time using human bone marrow–derived (hBM)-MSCs stored in LR-3T and LR-3T-5D.

The present study was approved by the Ethics Committee of Otsuka Pharmaceutical Factory, Inc. hBM-MSCs (male, 31 years, lot no. 0000527428; Lonza Walkersville, Inc., Walkersville, MD), lactated Ringer’s (LR) solution (Lactec® Injection), LR-3T (Cellstor-W), and LR-3T-5D (Cellstor-S) were used in this study. These solutions were supplied by Otsuka Pharmaceutical Factory, Inc., Tokushima, Japan. hBM-MSCs were expanded and used according to previous reports (Nishimura *et al.* 2019).

The first experiment examined the effect of storage time on the aggregation and viability of hBM-MSCs stored in LR, LR-3T, or LR-3T-5D at 25°C. After centrifugation at 600 × *g* for 5 min, the cells were resuspended at a density of 5 × 10^5^ cells/mL in LR, LR-3T, or LR-3T-5D, and 500-μL aliquots of each cell suspension were transferred to low-cell adsorption 15-mL tubes (STEMFULL™, Sumitomo Bakelite Co. Ltd., Tokyo, Japan). The tubes were tightly capped and then stored at 25°C in an incubator for various times (0, 1, 3, 6, and 24 h). The total numbers of cells and cell aggregates were determined using a NucleoCounter® NC-200™ (ChemoMetec A/S, Allerød, Denmark). Cell aggregation was defined as an association of ≥5 cells (Figure 1). The cell aggregation rate (%) was calculated from the total number of cells counted. The cells were stained with trypan blue to assess viability. Data were analyzed using SAS 9.4 software (SAS Institute, Inc., Cary, NC).

**Figure 1.**
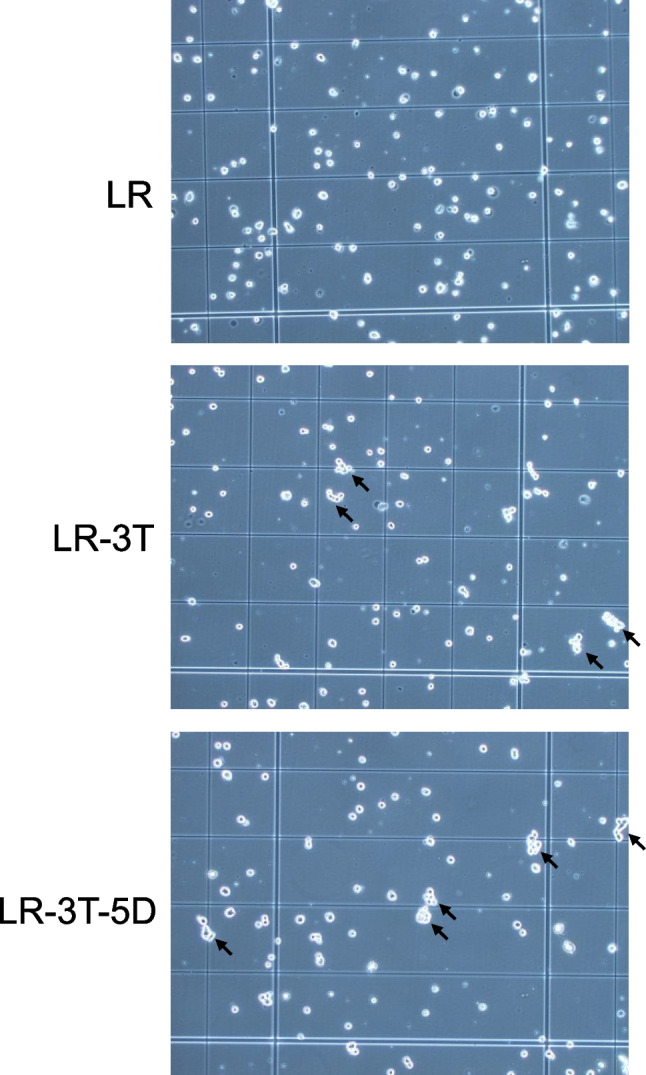
*Micrographs* of human bone marrow–derived stem cells (hBM-MSCs) after storage at 25°C for 24 h in lactated Ringer’s solution (LR), LR with 3% trehalose (LR-3T), or LR-3T with 5% dextran 40 (LR-3T-5D) (magnification 100×). *Arrows* indicate cell aggregates.

As shown in Figure 2*A*, there were no significant differences in cell aggregation rates after storage in each solution at 25°C for up to 6 h compared with before storage (Pre). However, the aggregation rate of hBM-MSCs stored in LR-3T or LR-3T-5D for 24 h was significantly higher (*p*<0.01) than the pre-storage rate. A comparison between solutions at each time point showed that storage in LR-3T-5D resulted in significantly higher aggregation than storage in LR-3T or LR at 6 h (*p*<0.01) and higher than storage in LR at 24 h (*p*<0.05). When stored at 25°C for 24 h, the viability of hBM-MSCs stored in LR was significantly lower (*p*<0.001) than the pre-storage rate, whereas the viability of cells stored in LR-3T or LR-3T-5D was comparable to the pre-storage rate (Figure 2*B*). In addition, the viability of hBM-MSCs stored in LR was significantly lower than that of cells stored in LR-3T-5D for 1, 3, or 24 h (*p*<0.05) and lower than that of cells stored in LR-3T for 24 h (*p*<0.05). It has been suggested that cells with low viability show low adhesion and proliferation in colony-forming assays (Fujita *et al.* 2021). Our observations may also indicate that a decline in cell viability during storage would be associated with low cell aggregation. Storage in LR-3T-5D at 25°C for 6 h resulted in more cell aggregation than when cells were stored in LR-3T. The difference between the two solutions is the presence or absence of dextran 40. The dextran 40 in LR-3T-5D inhibits the sedimentation of cells in bags or syringes and helps maintain a given concentration of cells at the time of administration (Fujita *et al.* 2020). However, dextran coatings reportedly enhance the adhesion of BM-MSCs to sponge scaffolds (Togami *et al.* 2014); thus, the adhesion-enhancing effect of dextran 40 may be responsible for the observed high degree of cell aggregation in LR-3T-5D at room temperature.

**Figure 2.**
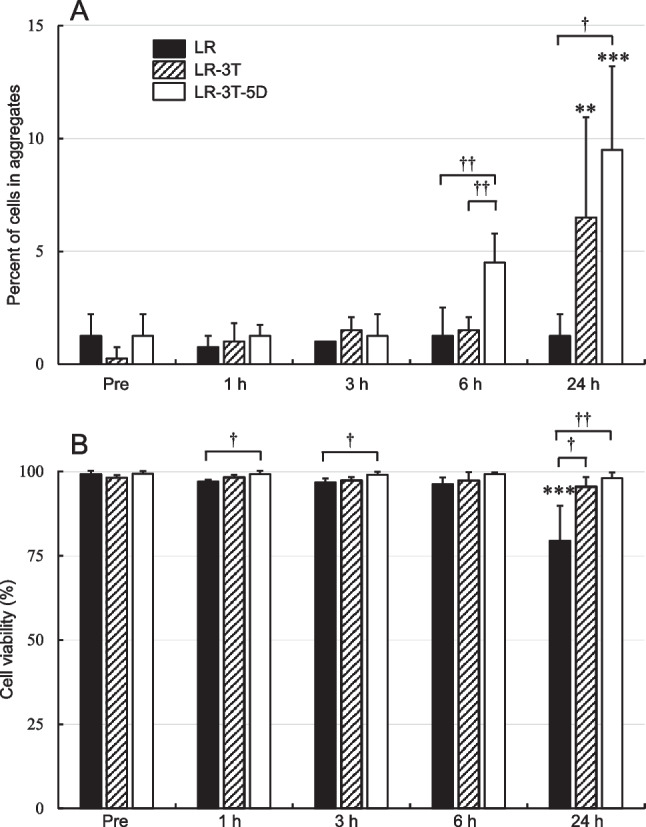
Aggregation rate (*A*) and viability (*B*) of hBM-MSCs after storage at 25°C for various times in LR, LR-3T, or LR-3T-5D. Data are presented as the mean ± SD (*n* = 4). Statistical analysis was performed using two-tailed Dunnett’s test vs. before storage (Pre): **p<0.01, ***p<0.001, and using two-tailed Tukey’s test: ^†^*p*<0.05, ^††^*p*<0.01.

The second experiment examined the effect of storage temperature on aggregation and viability of hBM-MSCs stored at 5 or 25°C for 24 h in LR-3T-5D, the solution in which the greatest amount of cell aggregation occurred in the first experiment. As described above, the cells were resuspended in LR-3T-5D at a density of 5 × 10^5^ cells/mL, and 500-μL aliquots of the cell suspension were transferred to separate tubes and stored for 24 h at 25°C and 5°C. As shown in Figure 3*A*, the aggregation rate of hBM-MSCs stored in LR-3T-5D for 24 h at 25°C was significantly higher (*p*<0.01) than the pre-storage rate, whereas the aggregation rate of hBM-MSCs stored in LR-3T-5D for 24 h at 5°C was similar to the pre-storage rate. The viability of hBM-MSCs stored in LR-3T-5D for 24 h at 5°C and 25°C was similar to the pre-storage viability (Figure 3*B*). The expression of membrane proteins such as cadherins may play an important role in MSC adhesion (Wuchter *et al.* 2007). The mechanism of storage-associated cell aggregation remains unclear, but cell aggregation may be inhibited due to the suppression of membrane protein function at low temperatures.

**Figure 3.**
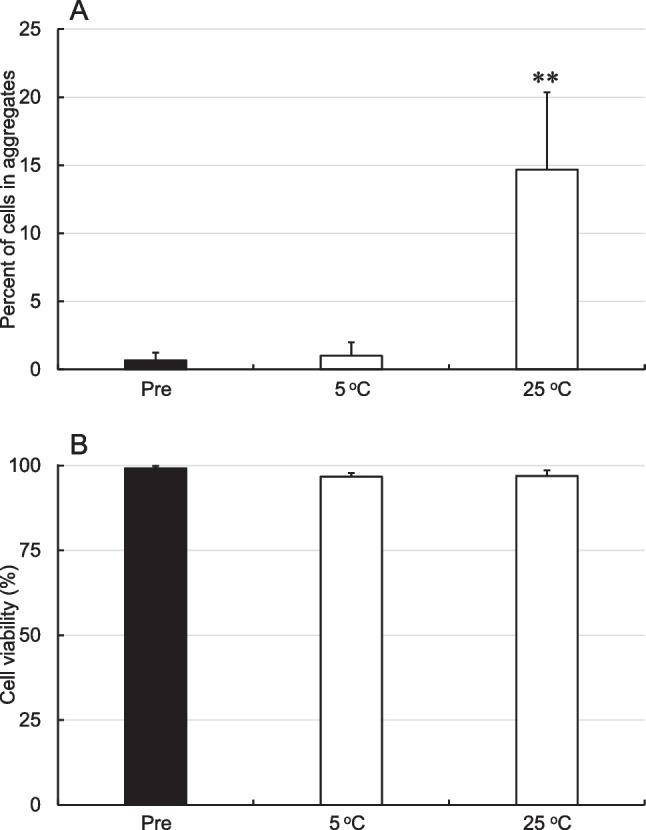
Aggregation rate (*A*) and viability (*B*) of hBM-MSCs after storage at 5 or 25°C for 24 h in LR-3T-5D. Data are presented as the mean ± SD (*n* = 3). Statistical analysis was performed using two-tailed Dunnett’s test vs. before storage (Pre): **p<0.01.

The third experiment examined the effect of trypsinization treatment time during cell collection on aggregation and viability of hBM-MSCs stored at 25°C for 24 h in LR-3T-5D. Immediately following cell collection from the flasks, the cells were trypsinized with trypsin/EDTA solution for 5 or 10 min at 25°C and resuspended in LR-3T-5D at a density of 5 × 10^5^ cells/mL. The cell suspensions (500 μL each) were transferred to separate tubes and stored for 24 h at 25°C. As shown in Figure 4*A*, when, the aggregation rate after storage of hBM-MSCs trypsinized for 10 min for cell collection was significantly lower than that of cells trypsinization for 5 min (*p*<0.05). There was no significant difference in cell viability, however (Figure 4*B*). Thus, cell aggregation was not inhibited due to a decrease in viability, as was the case when cells were stored at 25°C for 24 h in LR. A number of reports have indicated that trypsin treatment decreases the expression of surface proteins on MSCs (Garg *et al.* 2014; Tsuji *et al.* 2017; Nakao *et al.* 2019). High concentrations of trypsin were shown to suppress CXCR4 expression in BM-MSCs (Pervin *et al.* 2021). N-cadherin is required for the aggregation of HEK293T cells after trypsin detachment, and suppressing N-cadherin also suppresses aggregation (Tachibana 2019). We therefore believe that a trypsin treatment time of 10 min suppresses the function of surface proteins involved in adhesion, including cadherins, thereby also suppressing storage-associated aggregation.

**Figure 4.**
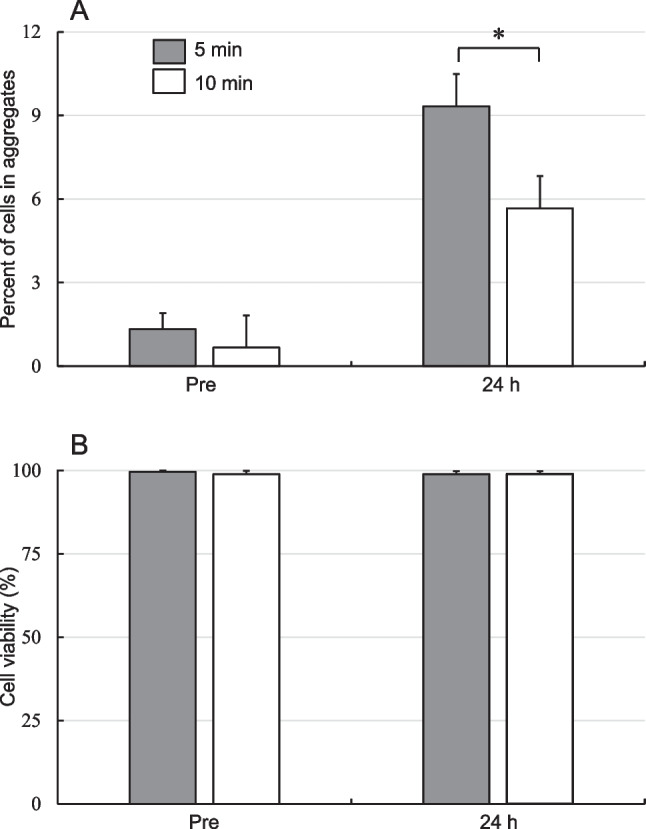
Aggregation rate (*A*) and viability (*B*) of hBM-MSCs collected by trypsinization for 5 or 10 min and stored in LR-3T-5D for 24 h at 25°C. Data are presented as the mean ± SD (*n* = 3). Statistical analysis was performed using two-tailed non-paired *t*-test: *p<0.05.

This study showed that storage of hBM-MSCs in LR-3T or LR-3T-5D for 24 h at 25°C is associated with high cell aggregation rates, although cell viability is maintained. In contrast, aggregation is suppressed when the cells are stored at 5°C. Furthermore, cell aggregation might be suppressed by prolonging the length of trypsinization from 5 to 10 min.

## Data Availability

The data that support the findings of this study are available from Otsuka Pharmaceutical Factory, Inc., but restrictions apply to the availability of these data, which were used under license for the current study and are therefore not publicly available. However, data are available from the corresponding author upon reasonable request and with permission of Otsuka Pharmaceutical Factory, Inc.
